# Effect of Cysteine on Methylglyoxal-Induced Renal Damage in Mesangial Cells

**DOI:** 10.3390/cells9010234

**Published:** 2020-01-17

**Authors:** Jae Hyuk Lee, Lalita Subedi, Sun Yeou Kim

**Affiliations:** 1 College of Pharmacy, Gachon University, #191, Hambakmoero, Yeonsu-gu, Incheon 21936, Korea; wogur6378@naver.com (J.H.L.); subedilali@gmail.com (L.S.); 2 Gachon Institute of Pharmaceutical Science, Gachon University, #191 Hambakmoe-ro, Yeonsu-gu, Incheon 21936, Korea; 3 Gachon Medical Research Institute, Gil Medical Center, Incheon 21565, Korea

**Keywords:** methylglyoxal, advanced glycation end products, l-cysteine, glyoxalase-I, diabetic nephropathy

## Abstract

Methylglyoxal (MGO), a highly reactive dicarbonyl compound, is a key precursor of the formation of advanced glycation end products (AGEs). MGO and MGO-AGEs were reportedly increased in patients with diabetic dysfunction, including diabetic nephropathy. The activation of glyoxalase-I (GLO-I) increases MGO and MGO-AGE detoxification. MGO-mediated glucotoxicity can also be ameliorated by MGO scavengers such as *N*-acetylcysteine (NAC), aminoguanidine (AG), and metformin. In this study, we noted that l-cysteine demonstrated protective effects against MGO-induced glucotoxicity in renal mesangial cells. l-cysteine prevented MGO-induced apoptosis and necrosis, together with a reduction of reactive oxygen species (ROS) production in MES13 cells. Interestingly, l-cysteine significantly reduced MGO-AGE formation and also acted as an MGO-AGE crosslink breaker. Furthermore, l-cysteine treatment accelerated MGO catabolism to D-lactate via the upregulation of GLO-I. The reduction of AGE formation and induction of AGE breakdown, following l-cysteine treatment, further supports the potential use of l-cysteine as an alternative for the therapeutic control of MGO-induced renal complications in diabetes, especially against diabetic nephropathy.

## 1. Introduction

Methylglyoxal (MGO) is a dicarbonyl compound produced by the degradation of carbohydrates [1]. Hyperglycemia with diabetic complications is the main cause of the excessive accumulation of MGO in the body [2,3]. Furthermore, MGO is a key precursor for the formation of advanced glycation end products (AGEs) [1]. AGEs are well-reported pathogenic factors in several human ailments including various diabetic complications such as diabetic neuropathy, nephropathy, retinopathy, etc. [4]. Cardiovascular and chronic kidney diseases (CKD) are considered as the major complications in diabetic patients that have been caused by MGO/MGO-AGEs [5,6]. Notably, increased MGO levels in diabetic patients are believed to contribute to vascular dysfunction and renal impairment [6]. There are increasing reports suggesting that chronic renal disease can be caused by excessive MGO accumulation and MGO-glyoxalase system inactivation [7,8,9]. Accordingly, MGO could be considered as a potential treatment target in diabetic nephropathy [8,10]. A previous study reported that higher MGO plasma levels were linked with disturbed glomerular filtration rates and diabetic chronic kidney disease [8]. Those cellular toxicities to renal cell lines by MGO and MGO-AGEs were reported to be regulated by mitogen-activated protein kinase (MAPK) signaling [11,12,13,14] suggesting that the inhibition of MAPK activation might prevent the renal cell death.

Aminoguanidine (AG), pyridoxamine, metformin, alagebrium (ALT-711), and *N*-acetylcysteine (NAC) are MGO scavengers [15,16,17,18] that have demonstrated potential effects in protecting cardiovascular as well as renal system against MGO-mediated toxicities [6]. NAC is a potent antioxidant, found in several dietary supplements that can induce protein phosphatase and reduce glutathione (GSH), neutralizing MGO to nonreactive and non-toxic forms such as hemithioacetal, s-lactoylglutathione, and D-lactate [18,19,20,21,22]. Yang et al. reported that NAC inhibited the MGO-induced AGE/RAGE expression in HaCaT cells [23]. Additionally, Zhou et al. reported that NAC improved the MGO-induced neurotoxicity in hippocampal neurons [24]. Collectively, NAC is a well-known candidate that lowers the oxidative damage in physiology; however, negative effects like vomiting, rash, and high fever limit its long-term use [25]. Interestingly, Yildiz et al. reported that the bio-availability of cysteine in erythrocytes is higher than that of NAC, suggesting that l-cysteine could dominate due to its biological activities over NAC [26]. Since NAC is the pro-drug of l-cysteine, and l-cysteine is one of the precursors of GSH, NAC/l-cysteine/GSH could be responsible for the modulation of the glyoxalase system and could play an important role in the regulation of MGO toxicities [21,27].

Since MGO is toxic to endothelial cells in the cardiovascular and renal systems, we hypothesized that the utilization of MGO scavengers to neutralize/inactivate MGO could be beneficial for the protection of kidney cells against MGO/MGO-AGE-mediated toxicities. Currently, there has been no report on the protective effect of l-cysteine in MGO and MGO-AGE-induced renal dysfunction. In this study, we aimed to explore the potency of l-cysteine against MGO-induced toxicity in renal mesangial cells. We also screened the potency of NAC, cystine, and l-cysteine against glucotoxicity including MGO toxicity, MGO-AGE formation, and MGO scavenging. l-cysteine showed the highest effectiveness to lower the glucotoxicity. Therefore, we select l-cysteine to further explore the underlying protective mechanism of l-cysteine against glucotoxicity.

## 2. Materials and Methods

### 2.1. Chemicals and Reagents

MGO, AG, 2′,7′-dichlorofluorescein diacetate (DCF-DA), sodium azide, l-cysteine, 3-(4,5-dimethylthiazol-2-yl)-2,5-diphenylterazolium bromide (MTT), and monoclonal anti-α-Tubulin antibody produced in mouse (α-Tubulin, cat no. T5168) were purchased from Sigma-Aldrich (St. Louis, MO, USA). Bovine serum albumin (RD tech, C0082-100), Dulbecco’s Modified Eagle’s Medium/Nutrient Mixture F-12 (DMEM/F-12) (WELGENE, Seoul, South Korea), Hyclone Dulbecco’s Modified Eagle Medium (DMEM) with high glucose (GE Healthcare, Chicago, IL, USA), FITC Annexin V Apoptosis Detection Kit I (BD Biosciences Pharmingen, San Diego, CA, USA) were purchased. Hoechst 33342 (Waltham, MA, USA) was purchased from Thermo Fisher Scientific. Alexa Fluor^®^ 555 Phalloidin (cat no. 8953S), p38 MAPK (cat no. 9212S), phospho-p38 MAPK (cat no. 9211S), SAPK/JNK (cat no. 9252S), phospho-SAPK/JNK (9251S), p44/42 MAPK (Erk1/2) (cat no. 9102S), phospho-p44/42 MAPK (Erk1/2) (cat no. 9101S), cleaved Caspase-3 (cat no. 9661S), and PARP (cat no. 9542S) were purchased from Cell Signaling Technology (Danvers, MA, USA). Glyoxalase I (cat no. sc-67351), SIRT1 (cat no. sc-15404), Bcl-2 (cat no. sc-492), Bax (cat no. sc-493) were obtained from Santa Cruz Biotechnology (Santa Cruz, CA, USA).

### 2.2. Cell Culture and Treatment

SV 40 MES13 (murine mesangial) and HEK 293 (human embryonic kidney) cells were purchased from the American Type Culture Collection (ATCC, Manassas, VA, USA). The cells were grown in DMEM/F-12 and DMEM with high glucose containing 10% FBS, 1% penicillin/streptomycin in a humidified atmosphere of 5% CO_2_ at 37 °C. For the main experiments, mesangial cells were treated with MGO at several concentrations (100, 500, and 1000 μM) for 24 h. l-Cysteine (0.1, 0.5, and 1.0 mM) and AG (1.0 mM) were added 1 h prior to MGO treatment.

### 2.3. Measurement of Cell Viability and Morphological Changes

MES13 and HEK 293 cells were seeded at 1.5 × 10^4^ and 5.0 × 10^4^ cells/well in 96-well plates and incubated for 24 h. The cells were pretreated with several concentrations (0.1, 0.5, and 1.0 mM) of l-cysteine and AG (1.0 mM) for 1 h, followed by treatment with MGO (500 μM) for 24 h. After incubation, the MTT (0.1 mg/mL) solution was added to each well and the cells were incubation for 2 h. Next, the medium was carefully removed and dimethyl sulfoxide (DMSO, Sigma-Aldrich, St. Louis, MO, USA) was added. The absorbance was measured at 570 nm using a microplate reader (Molecular Devices, San Jose, CA, USA). Cell morphological changes in mesangial cells were investigated using the IncuCyte Zoom imaging system (Essen Bioscience, Ann Arbor, MI, USA).

### 2.4. Measurement of LDH Production

Lactate dehydrogenase (LDH) production was measured using the Pierce LDH cytotoxicity assay kit (Thermo Scientific, Waltham, MA, USA) according to the manufacturer’s protocol. Briefly, cells were treated with test compounds. The conditioned medium (CM; 50 μL) from the treated cells was collected and transferred to a 96-well flat-bottom plate. Next, 50 μL of the reaction mixture was added to the CM-containing wells. The CM and working reagents were mixed well by placing the plate on a shaker for 30 min. The color change was evidenced by measuring the absorbance at 490 nm and 680 nm using a microplate reader (Molecular Devices, San Jose, CA, USA).

### 2.5. Measurement of Cell Apoptosis by Flow Cytometry

FITC/Annexin V Apoptosis Detection Kit I (BD Biosciences Pharmingen, San Diego, CA, USA) was used to determine apoptosis by flow cytometry according to the manufacturer’s protocol with slight modification. Briefly, 1.0 × 10^6^ cells were collected, washed with cold phosphate buffered saline (PBS) and the cells were resuspended in 1× binding buffer. Additionally, 5 μL of fluorescein isothiocyanate (FITC) Annexin V and 5 μL propidium iodide (PI) were added and the mixture was vortexed for proper mixing of the cells. Next, the mixture was incubated for 15 min in the dark at room temperature (25 °C) following the addition of 400 μL of 1× binding buffer to each sample. The prepared samples were analyzed using flow cytometry (FACSCalibur^TM^; Becton-Dickinson, San Jose, CA, USA) within 1 h.

### 2.6. Measurement of Intracellular ROS

Intracellular ROS scavenging activity of l-cysteine was evaluated by 2′,7′-Dichlorofluorescin diacetate (DCF-DA) staining as described previously with slight modification [28]. Briefly, 3.0 × 10^5^ mesangial cells were seeded in a 6-well plate. After incubation for 24 h, cells were pretreated with l-cysteine for 1 h, followed by incubation with MGO for 1 h. Cells were washed twice with PBS, then 20 μM DCF-DA was added. After incubation of cells for 20 min at 37 °C the cells were washed with PBS, photographed and measured using a JuLI live-cell imaging system (NanoEnTek, Seoul, Korea) and flow cytometry.

### 2.7. Measurement of D-lactate Production

D-lactate production was measured using the EnzyChrom^TM^ D-lactate assay kit (EDLC-100) (BioAssay Systems, Hayward, CA, USA) according to the manufacturer’s protocol. Briefly, cells were treated with test compounds and the CM from the treated cells was collected and stored for future use to perform D-lactate assay. First, 20 μL of standards and CM were transferred into the wells of a clear bottom 96-well plate. Reagent preparation was performed by mixing 60 μL assay buffer, 1 μL enzyme A, 1 μL enzyme B, 10 μL NAD and 14 μL MTT. Next, 80 μL of the working reagent was added to the wells containing standards and CM. The colorimetric changes were analyzed by measuring the absorbance at 565 nm using a microplate reader (Molecular Devices, San Jose, CA, USA).

### 2.8. Measurement of MGO-Derived AGE Formation

The MGO-AGE-formation assay was performed as described for the examination of inhibition in the several stages of the glycation process, according to the method of Kiho et al. [29] with slight modifications. Briefly, 5 mg/mL bovine serum albumin (BSA), 0.02% sodium azide were incubated with 5 mM MGO in the presence or absence of l-cysteine (0.1, 0.5, and 1.0 mM), AG (1.0 mM) in PBS (pH 7.4) for seven days, at 37 °C. The formation of AGEs was measured using fluorescence at excitation/emission wavelengths of 355/460 nm with a VICTOR ™ X3 multilabel plate reader (Perkin Elmer, Waltham, MA, USA).

### 2.9. Measurement of MGO-AGE Breakdown

MGO-AGE breaking ability was evaluated using the TNBSA (2, 4, 6-trinitrobenzene sulfonic acid) assay according to Habeeb et al. [16] with slight modifications. Briefly, MGO-AGE solution (1 mg/mL) was mixed with l-cysteine (0.1, 0.5, and 1.0 mM), AG (1.0 mM), and then incubated for 24 h, at 37 °C. After incubation, 4% NaHCO_3_ (pH 8.5) and 0.1% TNBSA were added to each Eppendorf tube for 2 h, at 37 °C. After incubation, 10% sodium dodecyl sulfate (SDS) and 1 N HCl were added. Next, the MGO-AGE breaking ability of l-cysteine was determined using the microplate reader at 340 nm (Molecular Devices, San Jose, CA, USA).

### 2.10. Western Blot Analysis

Mesangial cells were washed and harvested with ice-cold PBS (1×) and lysed in radio-immunoprecipitation assay buffer containing protease inhibitors. The cell lysates were centrifuged at 12,000× *g* for 20 min at 4 °C. Next, the supernatants were collected, and the protein concentration was evaluated by the Bradford Assay using bovine serum albumin (BSA) as the standard. Equal amounts of protein were separated using 6–15% density sodium dodecyl sulfate- polyacrylamide gel electrophoresis (SDS-PAGE) gels and transferred to PVDF membranes using the WSE-4040 HorizeBLOT 4M-R semi-dry transfer system (DAWINBIO, Gyeonggi-do, South Korea). Membranes were incubated with blocking buffer 5% skim milk in tris-buffered saline tween (TBST) for 1 h at room temperature and then incubated with the primary antibodies overnight at 4 °C. After washing, the membranes were incubated with the secondary antibody conjugated with horseradish peroxidase (HRP) for 1 h at room temperature. The protein signals were measured using a ChemiDoc XRS+ imaging system (Bio-Rad, Hercules, CA, USA).

### 2.11. Immunofluorescence Analysis

MES13 cells cultured on coverslips were incubated with several concentrations (0.1, 0.5, and 1.0 mM) of l-cysteine and AG (1.0 mM) for 1 h, followed by treatment with MGO (500 μM) for 24 h. After 24 h, the coverslips were washed three times with PBS and fixed in 10% formalin for 15 min at room temperature (25 °C). The fixed cells were then washed with PBS, dyed with Alex Fluor^®^ 555 Phalloidin to F-actin for 1 h and Hoechst 33342 for 15 min, and mounted with Fluoromount^TM^ aqueous mounting medium to fixation (St. Louis, MO, USA). After, they were measured under a laser scanning confocal microscope (Nikon A1+, Nikon, Tokyo, Japan). To measure F-Actin, random fields were selected in each experiment and several cells were imaged in each field. To evaluate F-Actin, NIS-Elements imaging software was used to quantify the fluorescence intensity.

### 2.12. Statistical Analysis

Statistical analyses were performed using GraphPad Prism version 5.00 (GraphPad Software, Inc., San Diego, CA, USA). The data are expressed as the mean ± SD. Statistical evaluations were analyzed using one-way ANOVA followed by Bonferroni’s post-test. A *p*-value of <0.05 was considered statistically significant.

## 3. Results

### 3.1. l-Cysteine Shows a Cytoprotective Effect against MGO-Induced MES13 Cell Toxicity

**Figure 1:**
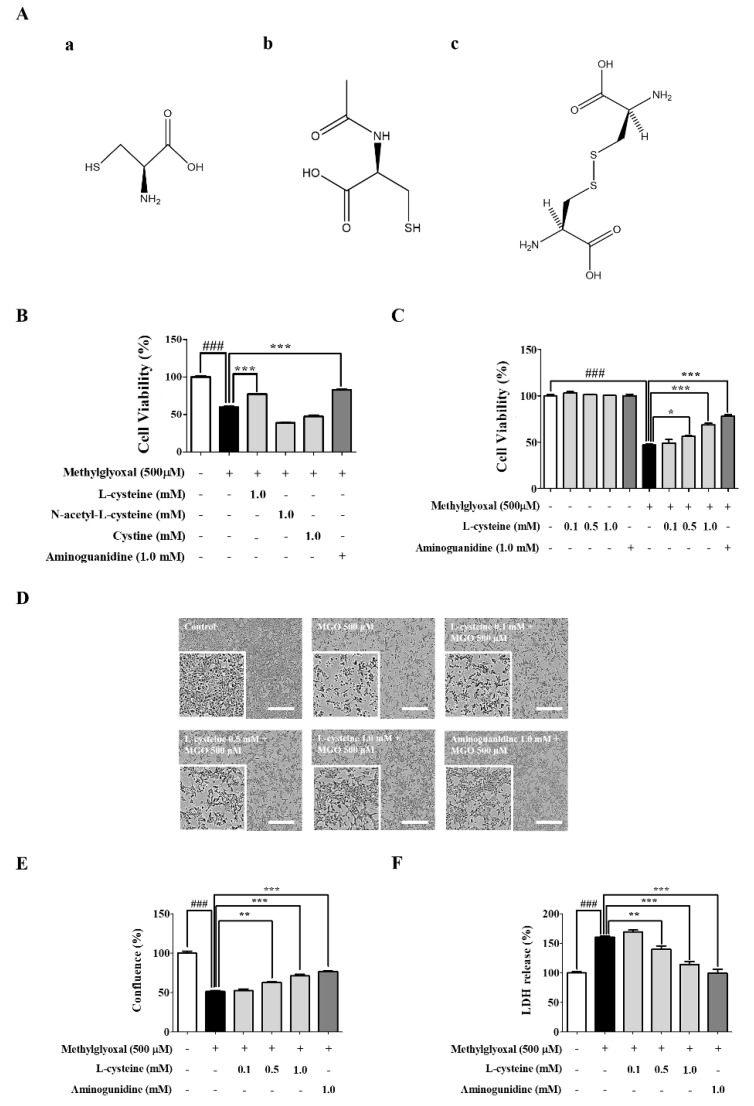
Effects of l-cysteine on methylglyoxal (MGO)-induced cell toxicity in MES13 cells. (**A**) Chemical structure of (**a**); l-cysteine, (**b**); *N*-Acetyl-l-cysteine (NAC), (**c**); Cystine. (**B**) Cell viability of MES13 cells treated with MGO (500 μM) and equal concentrations (1.0 mM) of l-cysteine, NAC, and cystine. (**C**) Cell viability of MES13 cells treated with MGO (500 μM) and various concentrations of l-cysteine (0.1, 0.5, and 1.0 mM) and analyzed using the MTT assay. (**D**) Representative photographs of l-cysteine pre-treatment protection against MGO-induced cytotoxicity. Scale bar indicates 500 μm. (**E**) Quantitative measurements of confluence were evaluated using IncuCyte Zoom imaging system. (**F**) MGO-induced LDH production was evaluated by LDH assay in MES13 cells. All data are presented as mean ± SEM. *n* = 3 (### *p* < 0.001 vs. Control, * *p* < 0.05, ** *p* < 0.01, *** *p* < 0.001 vs. MGO 500 μM).

**Figure 2:**
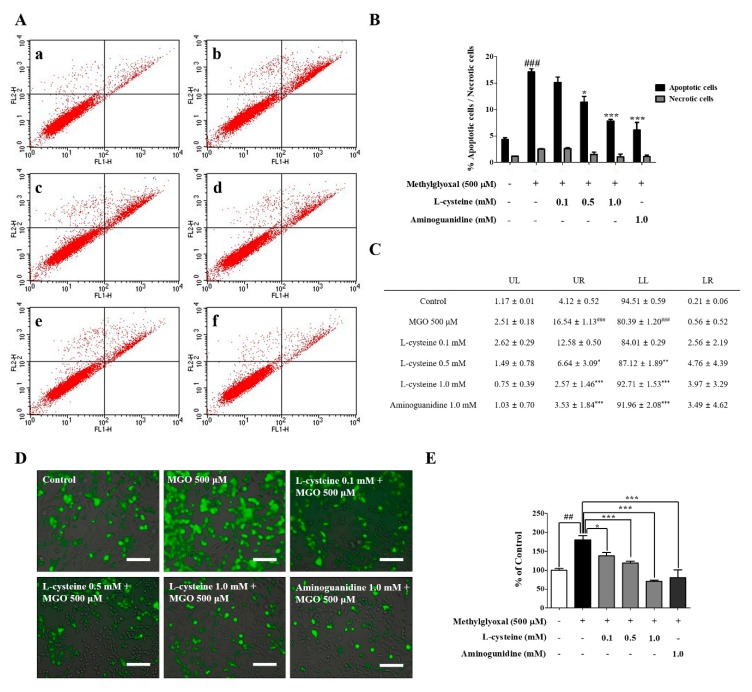
Effects of l-cysteine on MGO-induced apoptosis and reactive oxygen species (ROS) generation in MES13 cells. (**A**) Representative cytograms of Annexin V-fluorescein isothiocyanate (FITC) and propidium iodide (PI) staining of MGO-induced MES13 cells. Cells were pretreated with several concentrations l-cysteine for 1 h, then incubated with MGO (500 μM) for 24 h. After 24 h, the concentrations of viable (Annexin V-FITC and PI negative cells), early-stage apoptotic (Annexin V-FITC positive, PI negative cells), late-stage apoptotic (Annexin V-FITC positive, PI-positive cells), and necrotic (PI-positive cells) cells were analyzed by flow cytometry. (**a**) control; (**b**) 500 μM MGO; (**c**) MGO+l-cysteine (0.1 mM); (**d**) MGO+l-cysteine (0.5 mM); (**e**) MGO+l-cysteine (1.0 mM); (**f**) MGO+AG (1.0 mM) as a positive control. (**B,C**) Quantitative data of representative cytograms of Annexin V-FITC and PI staining. Percentage of control (LL), early-stage apoptotic (LR), late-stage apoptotic (UR), and necrotic cells (UL) as analyzed using BD CellQuest Pro software. (**D**) MES13 cells were pretreated with l-cysteine for 1 h, followed by 500 μM MGO for 1 h. Green fluorescence (ROS generation) from 2′,7′-Dichlorofluorescin diacetate (DCF-DA) was examined by JuLI live-cell imaging system. Scale bar indicates 500 μm. (**E**) Quantitative measurements of fluorescent intensity were evaluated using Image J software. All data are presented as mean ± SEM. *n* = 3 (## *p* < 0.01, ### *p* < 0.001 vs. Control, * *p* < 0.05, ** *p* < 0.01, *** *p* < 0.001 vs. MGO 500 μM).

Weiqand, T. et al. [30] and Cha, S.H. et al. [13] demonstrated that treatment of MGO (200–2000 μM) induced renal cell toxicity. Considering their results, we optimized the concentrations of MGO for the treatment in MES13 and HEK cell line in this study. We observed that MGO (0.1, 0.5, and 1.0 mM) treatment for 24 h induced cell toxicity and morphological changes in a dose-dependent manner (Figure S1A,B). Morphological changes were associated with reduced cell viability as measured by the MTT assay. The morphology of MES13 cells was captured using the IncuCyte Zoom imaging system (Essen Bioscience, Ann Arbor, MI, USA). From these results, we selected 500 μM concentration of MGO for further mechanism study. Similarly, Szwergold et al. demonstrated a range of concentration (340–920 μM) of carbonyl-scavenging amino acids and low concentration of MGO in blood plasma of birds [31]. This study gave us an idea for the treatment concentration of l-Cysteine as we hypothesized that l-cysteine might interact with MGO to lower MGO-induced toxicity. Altogether, ideas from previous studies and our concentration optimization experiments, we also selected 1mM concentration of l-cysteine for further mechanism study. The structures of l-cysteine, NAC, and cystine are shown in Figure 1A. MES13 cells were pretreated with 1.0 mM l-cysteine, NAC, and cystine for 1 h and then stimulated with MGO (500 μM) for 24 h. l-cysteine (0.5 mM and 1.0 mM) ameliorated MGO-induced cell toxicity and morphological changes (Figure 1B–D). MGO treatment significantly increased LDH production in MES13 cells. In the l-cysteine treated group, LDH production was significantly reduced in a dose-dependent manner (Figure 1E). Additionally, MGO-induced cell death was evaluated with the FITC Annexin V apoptosis detection kit using the flow cytometric technique. Accordingly, we determined the involvement of MGO in inducing necrosis and apoptosis. As shown in Figure 2A–C, treatment of the MES13 cells with MGO (500 μM) significantly increased the number of early apoptotic and late apoptotic cells. However, the increased early apoptotic and late apoptotic cells were significantly decreased by l-cysteine (0.5 mM and 1.0 mM) treatment in MES13 cells.

### 3.2. l-Cysteine Reduces MGO-Induced Intracellular ROS Generation

We investigated whether an accelerated generation of ROS by MGO can be controlled/lowered by l-cysteine treatment since it lowered cell death. ROS production was measured by DCF-DA staining and JuLI live-cell imaging system. As shown in Figure 2D,E, MGO induced an increased ROS generation, whereas l-cysteine pre-treatment significantly decreased the level of intracellular ROS in a dose-dependent manner in the MES13 cells.

### 3.3. l-Cysteine Downregulates MGO-Induced Cell Death and Its MAPKs Signaling Pathway

**Figure 3:**
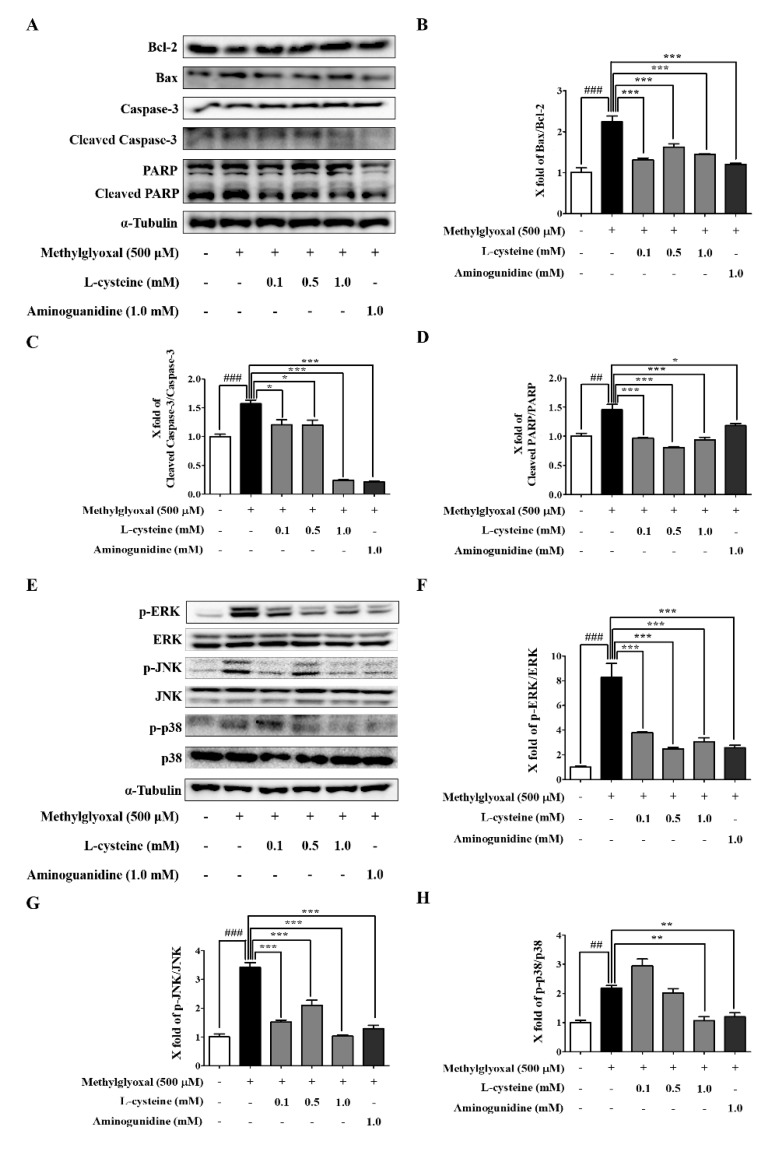
Effects of l-cysteine on apoptosis-related proteins and mitogen-activated protein kinase (MAPK) signaling pathway in MES13 cells. Cells were pretreated with l-cysteine for 1 h, followed by incubation with 500 μM MGO for 24 h. (**A**) The protein expression levels of Bax, Bcl-2, Caspase-3, and PARP were measured using western blot. (**B**–**D**) Bax/Bcl-2 ratio, Caspase-3, and PARP band intensity; α-tubulin was used as an internal control. (**E**) MES13 cells were pretreated with l-cysteine for 1 h, then incubated with 500 μM MGO for 1 h. The protein expression levels of MAPKs (extracellular signal-regulated kinase; ERK, c-Jun N terminal kinase; JNK, p38, and phosphorylated form) were examined by western blot. (**F**–**H**) p-ERK/ERK, p-JNK/JNK, and p-p38/p38 ratio and intensity; α-tubulin was used as an internal control. All data are presented as mean ± SEM. *n* = 3 (## *p* < 0.01, ### *p* < 0.001 vs. Control, * *p* < 0.05, ** *p* < 0.01, *** *p* < 0.001 vs. MGO 500 μM).

Using western blot analysis, we investigated the MGO-induced apoptosis and the signaling pathway of intracellular MAPKs (ERK, JNK, and p-38) in MES13 cells. MGO treatment for 24 h significantly induced the expression of proapoptotic proteins (Bax, Bcl-2, Caspase-3, and PARP) and the phosphorylation of MAP signaling proteins (ERK, JNK, and p38) compared to the control group (Figure 3). As shown in Figure 3A, as expected, l-cysteine pre-treatment significantly decreased the level of Bax/Bcl-2, cleaved Caspase-3/Caspase-3, and cleaved PARP/PARP in a dose-dependent manner. These results support our previous data where l-cysteine protected cells against MGO-induced toxicity (Figure 3A–D). In addition, pre-treatment with l-cysteine inhibited the phosphorylation of ERK, JNK, and p38, when all MAPKs were increased in MGO-induced MES13 cells (Figure 3E–H). Altogether, l-cysteine mediated the inhibition of MAPK activation, which could be responsible for lowering the expression of apoptosis-related proteins, as well as cell death.

### 3.4. l-Cysteine Regulates AGE Formation and Breakdown

**Figure 4:**
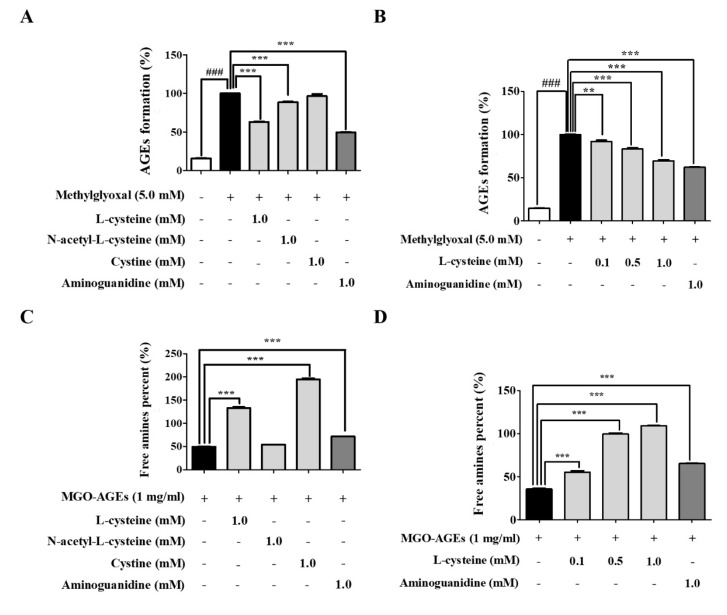
Effects of l-cysteine on the advanced glycation end product (AGE) formation and breakdown. (**A**,**B**) Effects of l-cysteine, NAC, and cystine on the in vitro formation of advanced glycation end products (AGEs) were evaluated using an AGE-formation assay; MGO-mediated AGE formation. Five mg/mL bovine serum albumin (BSA) and 0.02% sodium azide were incubated with 5 mM MGO in the presence or absence of each sample in PBS for 7 days. (**C**,**D**) AGE-breaking of preformed MGO-AGEs by l-cysteine, NAC, and cystine is shown as an increase in free amine groups compared to MGO-AGEs in the absence of l-cysteine, NAC, and cystine. All data are presented as mean ± SEM. *n* = 3 (### *p* < 0.001 vs. Control, ** *p* < 0.01, *** *p* < 0.001 vs. MGO 5.0 mM, MGO-AGEs 1 mg/mL).

We performed an AGE-formation assay by evaluating fluorescence with a VICTOR ™ × 3 multilabel plate reader (Perkin Elmer, Waltham, MA, USA). As shown in Figure 4A,B, MGO-BSA incubation significantly increased AGE formation. Under this condition, we investigated the inhibitory effects of l-cysteine, NAC, and cystine on MGO-BSA-induced AGE formation. l-cysteine treatment significantly inhibited the formation of MGO-AGEs in a dose-dependent manner (Figure 4A,B). In addition, l-cysteine, NAC, cystine, and AG were incubated with preformed MGO-AGE solution (1 mg/mL) for 24 h. MGO-AGE treatment significantly reduced free amine, compared to the control (control; 100 ± 1.25, MGO-AGEs; 49.23 ± 1.07). l-cysteine and cystine significantly restored MGO-AGEs free amine to a percentage of 132.91 ± 3.32 and 194.76 ± 3.57, respectively, at a concentration of 1.0 mM (Figure 4C). This result suggests that l-cysteine and cystine treatment significantly increase the breakdown of MGO-AGEs in a dose-dependent manner (Figure 4D and Figure S2D). Our results showed that l-cysteine and cystine increased the degradation of AGEs in MGO-induced glycated AGEs, and hence, these substances could be alternatives for the treatment of diabetic complications where MGO and MGO-AGEs are the key players.

### 3.5. l-Cysteine Prevents MGO-Induced Sirt1 and GLO-I Expression

**Figure 5:**
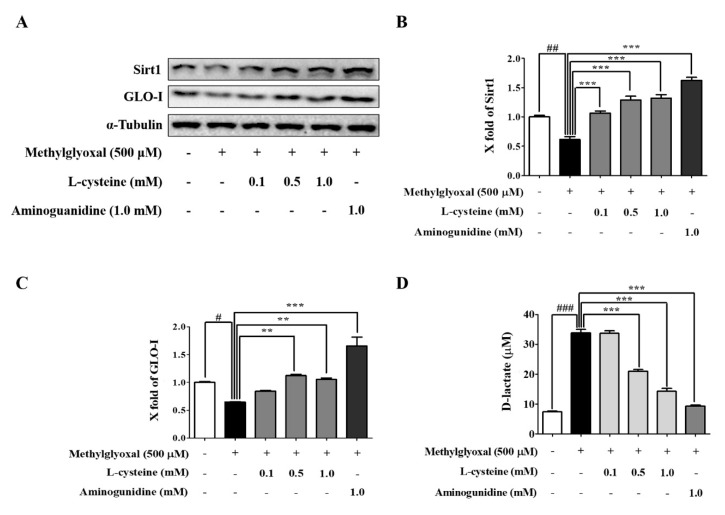
Effects of l-cysteine on glyoxalase system-related protein and D-lactate production in MGO-induced MES13. Cells were pretreated with l-cysteine (0.1, 0.5, and 1.0 mM) for 1 h, followed by incubation with 500 μM MGO for 24 h. (**A**) The protein expression levels of Sirt1 and GLO-I were evaluated by western blot. (**B**,**C**) Sirt1 and GLO-I band intensity; α-tubulin was used as an internal control. (**D**) Quantitative levels of D-lactate were shown in MES13 cells. All data are presented as mean ± SEM. *n* = 3 (# *p* < 0.05, ## *p* < 0.01, ### *p* < 0.001 vs. Control, ** *p* < 0.01, *** *p* < 0.001 vs. MGO 500 μM).

We performed western blotting to investigate whether MGO induced Sirt1 and GLO-I in MES13 cells. MGO treatment for 24 h significantly decreased the protein expression of Sirt1 and GLO-I. This was restored in the l-cysteine treated group in a dose-dependent manner, suggesting that l-cysteine not only protects MES13 cells from MGO-induced toxicity via inhibition of apoptotic and oxidative cascades but also through the activation of the Sirt1/glyoxalase system (Figure 5A–C).

### 3.6. l-Cysteine Accelerates MGO Catabolic Process to D-lactate

We investigated the level of D-lactate by using the EnzyChrom^TM^ D-lactate (Hayward, CA, USA) assay kit. MGO treatment significantly increased D-lactate in MES13 cells. In the l-cysteine treated group, D-lactate was significantly reduced in a dose-dependent manner (Figure 5D).

### 3.7. l-Cysteine Shows Cytoprotective Effects against MGO-Induced HEK 293 Cell Toxicity

**Figure 6:**
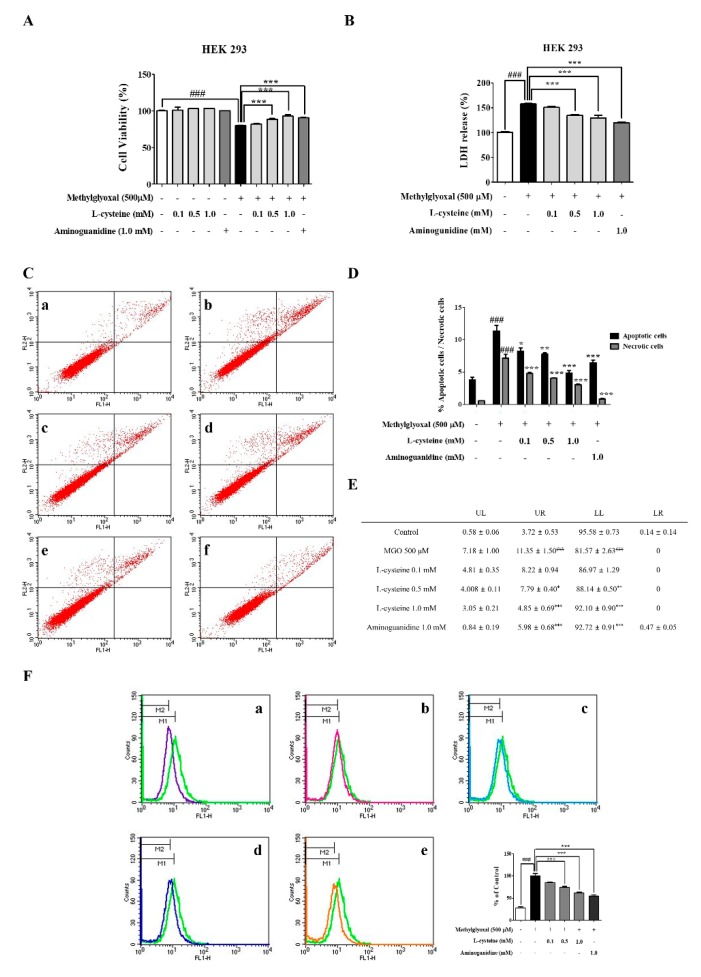
Effects of l-cysteine on MGO-induced cell toxicity and ROS generation in HEK 293 cells. (**A**) Cell viability of HEK 293 cells with MGO (500 μM) and various concentrations of l-cysteine (0.1, 0.5, and 1.0 mM) and analyzed using the MTT assay. (**B**) MGO-induced LDH production was evaluated by LDH assay in HEK 293 cells. (**C**) Representative cytograms of Annexin V-FITC and PI staining of MGO-induced HEK 293 cells. Cells were pre-treated with several concentrations l-cysteine for 1 h, then incubated with MGO (500 μM) for 24 h. After 24 h, the concentrations of viable (Annexin V-FITC and PI negative cells), early-stage apoptotic (Annexin V-FITC positive, PI negative cells), late-stage apoptotic (Annexin V-FITC positive, PI-positive cells), and necrotic (PI-positive cells) cells were analyzed by flow cytometry. (**a**) control; (**b**) 500 μM MGO; (**c**) MGO+l-cysteine (0.1 mM); (**d**) MGO+l-cysteine (0.5 mM); (**e**) MGO+l-cysteine (1.0 mM); (**f**) MGO+AG (1.0 mM) as a positive control. (**D**,**E**) Percentage of control, early-stage apoptotic, late-stage apoptotic, and necrotic cells as analyzed by flow cytometry. (**F**) MES13 cells were pretreated with l-cysteine for 1 h, followed by 500 μM MGO for 1 h. Green fluorescence (ROS generation) from DCF-DA was examined by FAC analysis. Quantitative measurements of fluorescent intensity were evaluated using an FAC analysis system. (**a**) control vs 500 μM MGO; (**b**) 500 μM MGO+ l-cysteine (0.1 mM); (**c**) MGO+l-cysteine (0.5 mM); (**d**) MGO+l-cysteine (1.0 mM); (**e**) MGO+ AG (1.0 mM) as a positive control. All data are presented as mean ± SEM. *n* = 3 (^###^ *p* < 0.001 vs. Control, * *p* < 0.05, ** *p* < 0.01, *** *p* < 0.001 vs. MGO 500 μM).

We investigated the cytotoxicity and LDH production by MTT assay and Pierce LDH assay kit (Thermo Scientific, Waltham, MA, USA). HEK 293 cells were pretreated with several concentrations (0.1, 0.5, and 1.0 mM) of l-cysteine. l-cysteine (0.5 mM and 1.0 mM) ameliorated MGO-induced cell toxicity and LDH production was significantly reduced in a dose-dependent manner (Figure 6A,B). Additionally, MGO-induced cell death was measured with the FITC Annexin V apoptosis detection kit using the flow cytometric technique. As shown in Figure 6C–E, treatment of the HEK 293 cells with MGO (500 μM) significantly increased the number of early apoptotic and late apoptotic cells. However, the increased early apoptotic and late apoptotic cells were significantly decreased by l-cysteine (0.5, and 1.0 mM) treatment in HEK 293 cells.

### 3.8. l-Cysteine Reduces MGO-Induced Intracellular ROS Generation in HEK 293 Cells

We investigated the ROS generation by DCF-DA staining and flow cytometer (FACSCalibur^TM^; Becton-Dickinson, San Jose, CA, USA) analysis. As shown in Figure 6F, MGO-induced an increased ROS generation, whereas l-cysteine pre-treatment significantly decreased the level of intracellular ROS in a dose-dependent manner in the HEK 293 cells.

### 3.9. l-Cysteine Restores MGO-Induced Cytoskeletal Protein Damage in MES13 Cells

**Figure 7:**
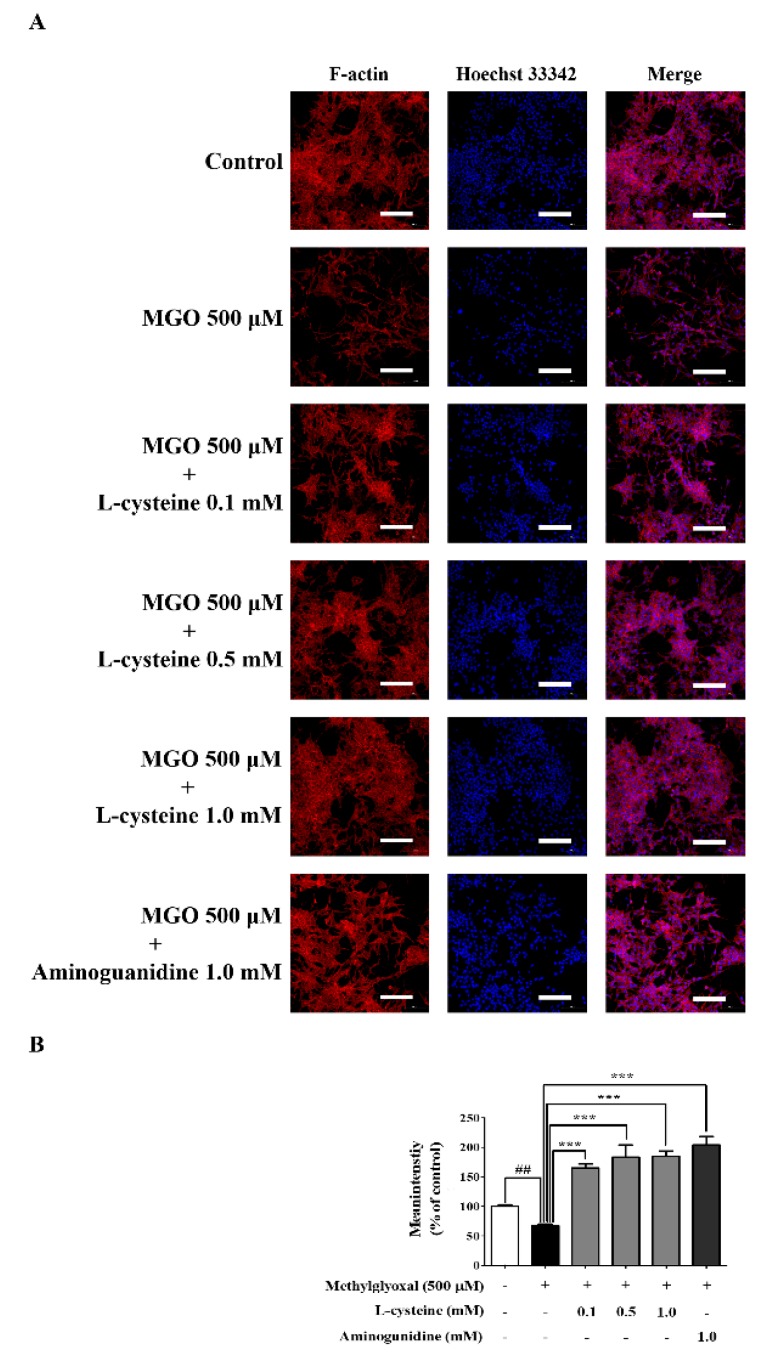
Effects of l-cysteine on MGO-induced cytoskeletal proteins in MES13 cells. (**A**) MES13 cells were pretreated with l-cysteine for 1 h, followed by 500 μM MGO for 1 h. The cells were stained with Hoechst 33342 (blue) and F-actin (Red) Alexa Fluor 555^®^ Phalloidin, and observed using confocal microscopy. The blue color indicated DAPI (nuclear) staining. Scale bar indicates 100 μm. (**B**) Quantitative measurements of F-actin fluorescence intensity were evaluated using NIS-Elements imaging software. Scale bar indicates 10 μm. All data are presented as mean ± SEM. *n* = 3 (^##^ *p* < 0.01 vs. Control, *** *p* < 0.001 vs. MGO 500 μM).

We investigated the change of cytoskeletal protein by immunofluorescence analysis (IF) and confocal microscope. As shown in Figure 7, MGO-induced a decreased F-actin intensity, whereas l-cysteine pre-treatment significantly increased the level of F-actin intensity in a dose-dependent manner in the MES13 cells.

## 4. Discussion

MGO and MGO-mediated AGEs are key toxicants involved in renal complications either in diabetic conditions or independent of diabetes [19]. Activation of the glyoxalase system through the interaction with GLO-I/II and glutathione was considered a solution for the prevention and treatment of such toxicity. However, this solution is limited in conditions where the glyoxalase system is less active and less enzymes or glutathione is available. Boollong et al. recently suggested that highly reactive MGO can interact with the cysteine residue present in Keap-1 protein to form a complex called MICA [32]. This notion provided a strong clue that, the formation of a complex between MGO and cysteine could prevent the reactive MGO-mediated cellular toxicity [32]. In our study, we established that treatment with l-cysteine can reduce the glucotoxicity induced by MGO in renal MES13 cells by lowering oxidative stress, AGE formation, and cell death.

It is well established that MGO-AGEs increase ROS production/oxidative stress and cell death in several endothelial cells [11,12,13,14]. This study demonstrated that MGO may induce MES13 cell death. Cell death/altered glomerular cell functioning might directly affect the glomerular filtration rate, a crucial dysfunction of the kidney. Therefore, downregulation of MGO-induced glomerular cell toxicity is necessary. Under this condition, we observed that l-cysteine pre-treatment significantly ameliorated the MGO-mediated MES13 and HEK 293 cell toxicity suggesting that l-cysteine might prevent renal cells from undergoing cell death induced by MGO toxicity. In addition, it was confirmed through the IncuCyte Zoom imaging system that the morphology and cytoskeleton of mesangial cells were damaged via MGO induction, and that this damage was recovered by l-cysteine treatment. This result supported our previous finding where MGO demonstrated cell toxicity in human kidney (HEK), renal epithelial (LLC-PK1), and rat mesangial cells in diabetic nephropathy [11,12,13,14]. The significant inhibition of apoptotic and necrotic cells following l-cysteine treatment was further verified by the significant inhibition of apoptotic protein expression including Bax/Bcl2, cleaved Caspase3/Caspase-3, cleaved PARP/PARP, etc. Our finding supports the fact that MGO might interact with cysteine forming an MGO-cysteine complex. This process prevents the reaction of MGO with other proteins and amino acids, resulting in cell survival. These results support the previous finding that MGO interacts with l-cysteine to form a complex [33]. Previously, Chumsae, C. et al. and Rabbani, N. et al. reported that MGO could interact with arginine and lysine to form AGEs (argipyrimidine, CEL, and MOLD); however, the interaction with l-cysteine and MGO, and their further consequences are not well reported [34,35]. In this study, we observed that the higher the l-cysteine treatment concentration, the higher the amount of MGO molecules that can interact and remain bound as a complex, and only free MGO molecules remained to induce cytotoxicity and oxidative damage in MES13 cells. Based on our study, we reported the induction of MAPK signaling and ROS production by MGO in renal cells, [11,36] the same effect observed in MES13 cells in this study. Interestingly, the toxic effect of MGO is significantly reversed following l-cysteine treatment. This result further suggests that the l-cysteine-MGO interaction reduced the MAPK signaling activation and ROS production, and this could explain the reduced cell death and damage. Similarly, this could be possible if MGO and l-cysteine react and form an irreversible complex [37]. Moreover, the protective effect of l-cysteine and AG treatment (1 mM) against MGO toxicity demonstrated almost similar potency in MES13 cells [11]. Overall, this implied that the l-cysteine treatment could be an alternative to AG for the treatment of renal impairment where MGO itself or MGO-derived AGEs are key players.

The excessive productions of MGO/MGO-AGEs are alarming factors in diabetes-mediated complications, especially in diabetes-mediated nephropathy where MGO/MGO-AGEs are noticed to be a key reasons behind the complications [8]. MGO breakdown or MGO entrapment presents an alternative treatment strategy. As MGO is a key precursor of AGE formation, incubation with l-cysteine not only lowered the MGO-AGE formation but also increased MGO-AGE breakdown. Both these conditions lower the level of AGEs and subsequently the AGE-mediated glucotoxicity. l-cysteine (1 mM) demonstrated similar potency to that of AG (1 mM) for the inhibition of AGE formation. However, even 0.5 mM of l-cysteine showed higher potency to increase AGE breakdown in comparison to 1 mM of AG as a positive control, suggesting the superior potential of l-cysteine to induced MGO-AGE breakdown. Furthermore, cystine was more effective than cysteine in AGE breakdown, and cysteine demonstrated a significant effect on the inhibition of AGE production. Michael A et al. reported that the inhibition of AGEs by strongly nucleophilic amino acids (e.g., cysteine and histidine) helps reduce diabetic and age-related complications. Since cysteine has a greater potency to inhibit AGEs, it could be an alternative for preventing diabetes-related complications via reduced AGE formation [38]. In the case of cystine, LR-20, a cystine derivative, demonstrated very high post-amadori inhibitory and metal chelation properties, but with low to moderate carbonyl scavenger properties. Therefore, the suppression of AGE generation by cystine is weaker than cysteine [39]. Overall, the higher potential of cysteine in depressing the formation of AGEs could prevent MGO/MGO-AGE-related complications, while the greater ability of cystine on AGE breakdown could act therapeutically to ameliorate the MGO/MGO-AGE-related complications. Further studies could explore the potential effect of cystine on depressing diabetes mediated complications in the near future. Additionally, l-cysteine could be a topic for further investigation as it not only lowered the MGO-mediated cellular toxicity but also controlled the AGE formation and breakdown, as required to manage diabetic nephropathy. MGO-mediated toxicity can be lowered in two major ways, either by inducing MGO breakdown or by MGO inactivation through MGO entrapment [19,40]. MGO breakdown is normally caused by the glyoxalase system, where GLO-I/II in the presence of GSH converts MGO to D-lactate. However, MGO entrapment/MGO inactivation could lower D-lactate conversion from MGO [19]. Previous studies reported the increased level of D-lactate in the urine of diabetes patients, indicating D-lactate as a marker for renal damage [41]. A higher level of GLO-I and a lower level of D-lactate in l-cysteine and AG treatment suggest that there might be less/no utilization of GLO-I, and hence less/no MGO gets converted to D-lactate. On the other hand numerous studies support the role of Sirt1 as a protective factor against MGO/-AGE-induced damage in cells and animal models [42,43,44]. Recent studies reported that Sirt1 can modulate MGO scavenging by promoting the expression of GLO-I in MGO-exposed mice [44]. In our study, we observed a similar result where activation of GLO-I was accompanied by Sirt1 activation following AG/l-cysteine treatment. Moreover, it can suggest that AG/l-cysteine-mediated activation of Sirt1/GLO-I might also participate their cytoprotective effects against MGO/MGO-AGE-induced toxicity either by lowering MGO-AGE formation or inducing the breakdown of MGO-MGO-AGEs. Additionally, inhibition of lactate dehydrogenase (LDH), released by the damaged cells following l-cysteine treatment, supports the protective effect of l-cysteine over MGO, possibly through MGO inactivation/entrapment.

In the previous study, Liang, Y.J. et al. reported that treatment of human embryonic kidney cells (HEK 293) with glyceraldehyde-AGEs/high glucose caused a pronounced upregulation/activation of RAGE/NF-κB pathway responsible for cell toxicity [45,46]. In our study, we also observed the similar toxicity of MGO while it was significantly ameliorated by l-cysteine treatment. Namely, treatment of l-cysteine could be an alternative solution for MGO-induced cell toxicity in human kidney cells. Therefore, this new experiment further supports the protective effect of l-cysteine cells on MGO-induced glucotoxicity not only in mouse but also in human kidney cells.

## 5. Conclusions

In this study, MGO-induced glucotoxicity in renal mesangial cells (mouse and human) was significantly ameliorated by l-cysteine treatment by lowering the oxidative stress and apoptotic cascades. We believe that l-cysteine can be a superior option for the inactivation of MGO and its complications in renal cell lines. Therefore, the present work may provide a basal evidence for the treatment of diabetic nephrotoxicity with l-cysteine. Further research in animal models and clinical studies are essential to illustrate the role of l-cysteine as a potential candidate against MGO/MGO-AGE-mediated diabetic complications, especially in diabetic nephropathy.

## Supplementary Materials

The following are available online at https://www.mdpi.com/2073-4409/9/1/234/s1, Figure S1: Effects of NAC and cystine on MGO-induced cell toxicity in MES13, Figure S2: Effects of NAC and cystine on the AGE formation and breakdown.
