# Supplementary material for: Effect of Cysteine on Methylglyoxal-Induced Renal Damage in Mesangial Cells
Source: Cells. 2020 Jan 17;9(1):234. doi: 10.3390/cells9010234 (PMC7016887; doi:10.3390/cells9010234)
Supplement: Supplementary file 1 [file cells-09-00234-s001.pdf]

Supplementary Materials

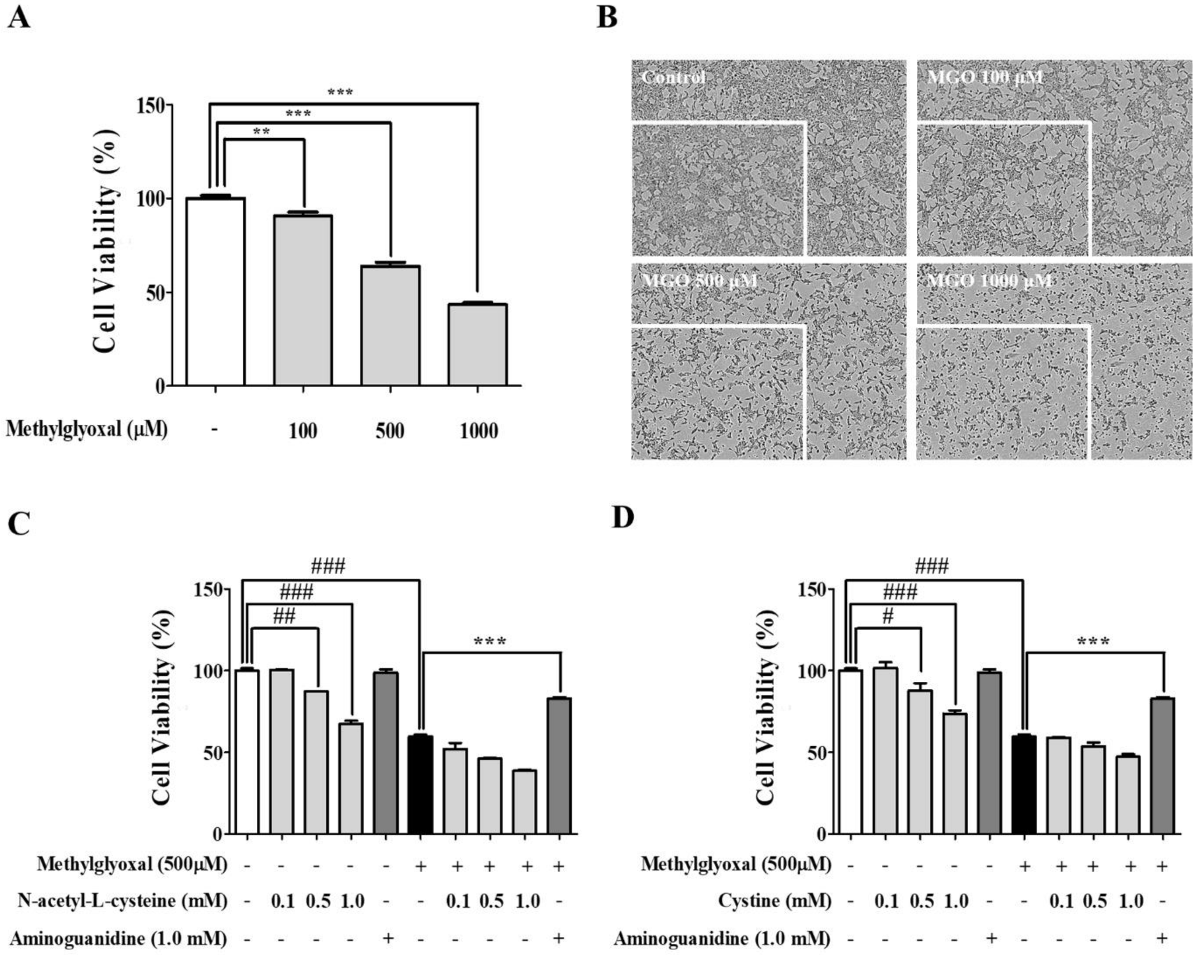

Figure S1. Effects of NAC and cystine on MGO-induced cell toxicity in MES13. (A, B) Cell viability and representative experiment photographs of MES13 treated with MGO (100, 500, and 1000 μM) for 24 h. (C, D) Cell viability of MES13 treated with MGO (500 μM) and various concentrations of NAC, cystine (0.1, 0.5, and 1.0 mM) and analyzed using the MTT assay. All data are presented as mean ± SEM. N = 3 (#p < 0.05, ##p < 0.01 vs. Control, ***p < 0.001 vs. MGO 500 μM).

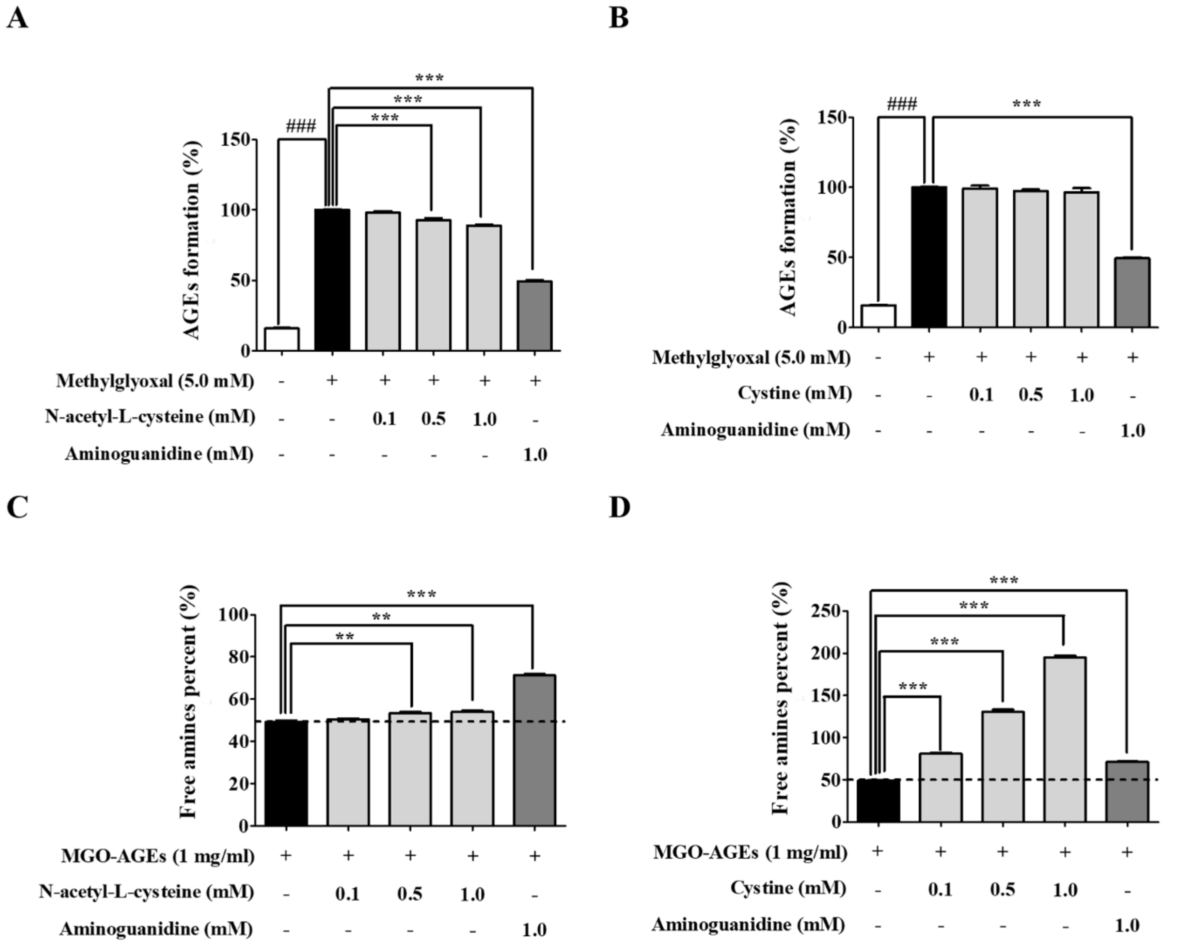

Figure S2. Effects of NAC and cystine on the AGEs formation and breakdown. (A, B) Effects of NAC and cystine on the in vitro formation of advanced glycation end products (AGEs) were assessed using an AGEs formation assay; MGO-mediated AGEs formation. BSA (5 mg/ml) and 0.02% sodium azide were incubated with 5 mM MGO in the presence or absence of each sample in PBS for 7 days. (C, D) AGEs-breaking of preformed MGO-AGEs by NAC and cystine is exhibited as an increase in free amine groups compared to MGO-AGEs in the absence of NAC and cystine. All data are presented as mean ± SEM. N = 3 (###p < 0.001 vs. Control, **p < 0.01, ***p < 0.001 vs. MGO 5.0 mM, MGO-AGEs 1 mg/ml).
